# A Cotinine in Freeze-Dried Urine Reference Material

**DOI:** 10.6028/jres.094.028

**Published:** 1989

**Authors:** Lane C. Sander, Gary D. Byrd

**Affiliations:** National Institute of Standards and Technology, Gaithersburg, MD 20899

**Keywords:** cotinine, cotinine Perchlorate, GC-MS, passive smoking, side stream smoke, standards, tobacco

## Abstract

A cotinine in freeze-dried urine reference material (RM 8444) was prepared at three concentrations: (1) a “blank” level typical of nonsmokers with no exposure to cigarette smoke, (2) a “low” level corresponding to nonsmokers with passive exposure to side-stream smoke, and (3) a “high” level typical of smokers. Low- and high-level materials were prepared gravimetrically from pooled urine by the addition of appropriate amounts of cotinine Perchlorate. Cotinine was determined by GC-MS using cotinine-*d*_3_) as an internal standard. No evidence for sample inhomogeneity was observed. This reference material will fulfill a need for a urine-based standard to assist in the validation of field methods used for assessing exposure to cigarette smoke.

## 1. Introduction

The correlation of adverse health effects with cigarette smoking has been based largely on self-reported data of the number of cigarettes smoked [1]. The validity of such data, however, may be limited due to denial, which is common among young smokers and announced quitters [2–4]. In addition, variables such as the “strength” of the cigarette (tar and nicotine levels), depth and frequency of inhalation, and duration of smoking affect the dose received by the smoker [2,5,6]. In studies involving passive exposure of nonsmokers to side-stream cigarette smoke, self-reported data are not applicable. Related problems exist in quantification of intake of smokeless tobacco products. Other studies have examined the exposure of infants to nicotine via milk from smoking mothers [7,8]. It is clear that an objective measure of exposure to cigarette smoke is needed to permit correlation of physiological changes with smoking.

A variety of compounds have been proposed as epidemiological markers for cigarette smoke exposure [2,9]. Cigarette smoke consists of a complex mixture of particles and gases containing appreciable quantities of nicotine, carbon monoxide, and hydrogen cyanide. These compounds and their metabolites are present in biological fluids (serum, saliva, urine) of smokers. Carbon monoxide is present as carboxyhemoglobin (COhb), and has a biological half-life of 2–4 h [10]. Because of this small half-life, COhb levels reflect only recent exposure to cigarette smoke. Environmental contributions of carbon monoxide further complicate interpretation and use of this marker. The half-life of nicotine is even shorter (30–80 min) [11] and thus reflects only transient exposure. Cumulative exposure is better correlated with serum thiocyanate levels (half-life 14 d) [12] resulting from transformation of hydrogen cyanide. Unfortunately, thiocyanate levels are also affected by diet, limiting its usefulness as an epidemiological marker. Perhaps the best single measure of cumulative exposure to tobacco products is given by cotinine [13]. Cotinine ((S)-1-methyl-5-(3-pyridinyl)-2-pyrrolidinone) is the major metabolite of nicotine. It has a biological half-life of approximately 20 h [14] and is found in the serum and urine of smokers. Cotinine levels are linearly and directly related to nicotine intake [5].

To assist in the validation of the accuracy of analytical methods for the determination of cotinine in urine, a freeze-dried urine containing cotinine was prepared for use as a reference material. This reference material, designated RM 8444, consists of three lots of freeze-dried urine containing different concentrations of cotinine: (1) an unspiked “blank” level, (2) a “low” level (approximately 50 ppb), and (3) a “high” level (approximately 500 ppb). These cotinine concentrations represent the levels that might be expected in nonsmokers with no exposure to cigarette smoke (or related nicotine containing products), nonsmokers with passive exposure to side-stream smoke, and smokers, respectively. The low- and high-level materials were prepared gravimetrically by spiking urine with known quantities of cotinine Perchlorate; the blank level material was unspiked. Because these reference materials are homogenous and well characterized, they are useful for verifying methods for the determination of cotinine in urine.

## 2. Experimental Section

### 2.1 Cotinine Standards

Because the free-base form of cotinine is hygroscopic, cotinine Perchlorate was used as the primary standard for this work. Cotinine Perchlorate was unavailable commercially and was synthesized from cotinine (Aldrich Chemical Co.^1^, lot 04112MT) and 70% perchloric acid, using the method of Jacob and Benowitz [15]. One g cotinine was distilled under vacuum at 225 °C. The product was dissolved in 15 mL isopropanol and added to 1 mL perchloric acid in 15 mL isopropanol. A white precipitate formed which dissolved upon swirling and reformed when cooled in an ice bath. The crystals were filtered and washed with portions of isopropanol and ethyl ether. After air drying, the product was dried under reduced pressure for 1 h.

Purity was determined by differential scanning calorimetry over the temperature interval 210–220 °C. The purity was determined to be 99.91 mol percent (melting point 218 °C). Also, the melting point was measured in a glass capillary, and crystals were observed to melt over the range 212–216 °C (cotinine Perchlorate mp = 218 °C [15]). No evidence of organic impurities was observed by liquid chromatography (LC) or gas chromatography (GC) analyses. A sample of the product was submitted to Galbraith Laboratories (Knoxville, TN) for elemental analyses. Duplicate determinations were made for carbon, hydrogen, nitrogen, chlorine, and oxygen (see table 1). Based on the molecular formula for cotinine Perchlorate, expected elemental percentages can be calculated. As shown in table 1, the measured composition compares favorably to the calculated values. On the basis of these characterizations, product purity was accepted to be greater than 99%, and no corrections for sample impurity were made.

Deuterated cotinine (cotinine-*d*_3_) was obtained from Cambridge Isotope Laboratories (Woburn, MA). The isotope purity determined by gas chromatography- mass spectrometry (GC-MS) was 98.7%. Corrections were made for the amount of undeuterated cotinine contributed, in all calculations. Because deuterated cotinine was obtained in the free-base form and readily absorbs water, the resultant weight gain makes it impossible to know the actual concentration of the internal standard spiking solution. The approximate concentration of this solution (12.4 ppm) was only used to make minor corrections to the response factors involving isotopic purity of the internal standard. The same internal standard solution was used throughout this study for both unknowns and response factor solutions. Response factors were determined using four independently prepared calibration solutions.

### 2.2 Preparation of Reference Materials

Cotinine reference materials were freeze dried. Samples from two freeze-drying runs were used for each of the low- and high-level spiked materials, and three runs for the blank material. Separate urine collections were made for the three materials. Donors (nonsmokers) were advised to avoid any exposure to tobacco smoke or caffeine for at least 48 h prior to the collection. Although caffeine does not interfere in GC-MS determinations of cotinine, the level of caffeine was reduced so as not to preclude the use of any liquid chromatographic methods (liquid chromatography was not used in the analysis of this reference material). Multiple urine pools were used (as opposed to a single urine pool) to avoid problems in storing the urine between the freeze-drying runs. Five mL aliquots of each of the urine samples were pipetted into 10-mL capacity serum cap vials. Excess material (a total in each case of about 8–9 L) was prepared to facilitate handling and to promote sample homogeneity.

Cotinine in urine materials were prepared gravimetrically using cotinine Perchlorate for the low-and high-level solutions. Cotinine was not added to the blank-level material. For low- and high-level materials, an appropriate amount of cotinine Perchlorate was weighed and dissolved in approximately 5-mL distilled water. This solution was transferred quantitatively to the pooled urine through a thistle tube using successive washings with water (total volume of solution added was approximately 125 mL). The final weight of spiked urine was recorded and the solution was stirred for at least 2 h before pipetting. Gravimetric values for the solutions are listed in table 2.

### 2.3 Freeze-Drying Procedure

Five-mL aliquots of the urine materials were pipetted into 10-mL serum cap vials using an automatic pipetter. About 1200 units were prepared at each concentration level. Prior to freeze-drying, the samples were frozen at −80 °C. The freezedrier was cooled to −50 °C (shelf temperature) in preparation to receive the samples (condenser at −70 °C). The two trays were rapidly transferred from the freezer to the freeze drier to minimize any wanning of the samples. Two temperature probes were used per tray, with probe placement at the center and edge of each tray. Immediately after transfer, the freeze drier was evacuated to start the freeze-drying process. Pressures were typically about 13 Pa (100 *µ*m Hg). During the initial pump down, sample foaming was observed in a fraction of the samples; foaming was most prevalent in samples near the edges of the trays (in “warm” samples).

The shelf temperature was set and maintained at −30 °C for the first 24 h period. This temperature was increased in roughly 15° increments every 12 h period. The temperature, as monitored by the probes, changed only slightly during the first 36 h. Freeze drying was considered complete when the shelf and probe temperatures were at ambient temperature, and the pressure was less than 13 Pa. The freeze-dried samples were sealed with the serum cap tops under a slight negative pressure. Total time to freeze dry each batch was about 72 h. Two freeze-drying runs were required for each reference material level.

### 2.4 Analysis by GC-MS

A solution of cotinine-*d*_3_ (approximately 12.4 ppm) was prepared in methanol and was ampouled for later use. This solution was used as an internal standard for both calibration standards (response factor solutions) and for the unknowns (urine reference materials). Four separate response factor solutions were prepared. Independent response factors were determined for each level. Separate response factors were calculated for *m/z* 98/101 and 176/179, and cotinine concentration values were determined based on both mass pairs.

Six vials from the low- and high-level materials were selected by a stratified random sampling scheme. Due to the very low cotinine level in the blank material, special procedures were required to estimate the cotinine in these samples (described below). The spiked urine materials were reconstituted by adding 5 mL of distilled water to each vial, followed by shaking and sonication for 60 s. Each sample was spiked with 50 *µL* of cotinine-*d*_3_ internal standard solution, followed by an addition of 10 drops of 10 *M* KOH. The samples were then extracted with 5 mL of methylene chloride. The methylene chloride layer was removed with a pipet and treated with 1–2 g of sodium sulfate. The methylene chloride was decanted into a centrifuge tube and evaporated to dryness under argon. The residue was dissolved in 50 *µ*L of methanol and analyzed by GC-MS.

**Figure 1:**
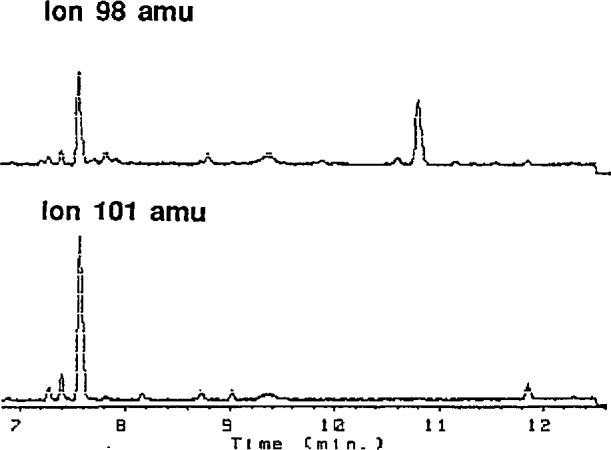
GC-MS determination of cotinine in reference material 8444. Ion chromatograms for mass fragments 98 (cotinine) and 101 (cotinine-*d*_3_) are shown.

A DB-210 250 *µ*m i.d. capillary column (J & W Scientific, Folsom, CA) was used for the GC-MS analysis of the urine extracts. This column is more polar than the more conventional DB-5 phase, and yields a better peak shape than the DB-5 phase. The following temperature program was used in the GC separation: 185 °C initial, linear ramp of 5 °C/min, final temperature of 225 °C. Between GC runs, the column was briefly ramped to 230 °C. Four masses were monitored: 176, 179, 98, and 101, corresponding to parent ions and major fragments from cotinine and cotinine-*d*_3_. A typical separation of a urine extract is illustrated in figure 1.

To obtain an estimate of the concentration of cotinine in the unspiked urine blank, 50 mL (10 vials) of urine were combined. Ten vials from the beginning, middle, and end of the blank preparation were analyzed. The uncertainty of this determination is high due to the small areas measured (signals were only a few times the noise level). The average result of the determinations is given in table 2.

### 2.5 Stability

Cotinine levels were again determined after a storage period of 160 d. During this time the materials were stored at ambient temperature for ~4 months, and at −20 °C for ~1 month. Cotinine levels were determined for low and high level samples as described above. The results of the determination are listed in table 2. Cotinine levels were found to be slightly lower than previously determined, however the values were still within the uncertainty of the measurement.

## 3. Results

### 3.1 Discussion

Careful examination of table 2 reveals that no significant difference exists between the values determined by gravimetry and GC-MS, for low- and high-level samples. Essentially all of the spiked cotinine (within measurement error) is recovered upon reconstitution of the sample. The cotinine concentrations calculated from gravimetric measurements are thus confirmed by independently determined values from GC-MS measurements. Cotinine concentrations determined after 160 d were similar to those determined originally, however small decreases in concentration were observed (see table 2). Possible sources for these decreases could include analyte degradation or irreversible adsorption onto particulate or other matter. Because the samples were stored at ambient temperature for approximately 4 months, it is possible that changes in cotinine concentration occurred at that time. Stability of this reference material should be enhanced by storage at or below 0 °C. Cotinine levels will be assessed periodically to assure that the concentrations remain within recommended ranges.

### 3.2 Recommended Values

The following recommended cotinine concentrations for the three reference materials are based only on the GC-MS determinations.
blank level: 0.8±0.3 ng/glow level: 
$52_{-5}^{+2}\;\text{ng}/g$high level: 
$485_{-10}^{+4}\;\text{ng}/g$These values are based on judgment, and reflect measurement uncertainties and possible sample instability. Average cotinine concentrations compare very favorably to the gravimetric values, and are within the error of the determination. It is clear that losses of the analyte due to freeze drying were negligible. No evidence of sample inhomogeneity was observed for any of the materials.

## Figures and Tables

**Table 1:** Elemental composition of cotinine Perchlorate^a^

Element	Calculated composition	Analysis #1	Analysis *#*2	Calculated purity (%)
Carbon	43.41	43.56	43.28	100.3, 99.7
Hydrogen	4.74	4.97	4.92	104.9, 103.8
Nitrogen	10.13	10.20	10.06	101.7, 99.3
Chlorine	12.81	12.60	12.88	98.4, 100.5
Oxygen	28.91	28.64	28.71	99.1, 99.3

a Elemental composition determinations performed by Galbraith Laboratories, Knoxville, TN.

**Table 2:** Gravimetric and GC-MS values for spiked cotinine in urine reference materials

Level	Weight solution	Weight cotinine Perchlorate	Cone. cotinine^a^ gravimetric	Cone. cotinine^a^ (original)	Cone. cotinine^a^ (160 d)
blank	8648 g			0.8±0.3 ng/g	
low	6808 g	0.593 mg	55.47 ng/g	56 ± 2 ng/g	52± 3 ng/g
high	7361 g	5.662 mg	489.88 ng/g	491 ± 6 ng/g	485±12 ng/g

a Cotinine concentration values are corrected for the weight of the Perchlorate salt.

## Footnotes

1 Certain commercial equipment, instruments, or materials are identified in this report to specify adequately the experimental procedure. Such identification does not imply recommendation or endorsement by the National Institute of Standards and Technology, nor does it imply that the materials or equipment identified are necessarily the best available for the purpose.

## References

[1] Haley NJ, Axelrad CM, Tilton KA (1983). Am J Public Health.

[2] Hill P, Haley NJ, Wynder EL (1983). J Chron Dis.

[3] Allen P, Lundi B, Westling H (1976). Psychopharmacology.

[4] Gillies PA, Wilcox B, Coates C, Kristmundsdotir F, Reid DJ (1982). J Epid and Commun Health.

[5] Galeazzi RL, Daenens P, Gugger M (1985). J Clin Pharmacol.

[6] Hill P, Marquardt H (1980). Clin Pharmacol Ther.

[7] Luck W, Nau H (1985). J Pediatrics.

[8] Luck W, Nau H (1984). Br J Clin Pharmacol.

[9] Vesey CJ, Saboojee Y, Cole PV, Russell MAH (1982). Brit Med J.

[10] Wald N, Howard S, Smith MG, Bailey A (1975). Thorax.

[11] Issac PG, Rand MJ (1972). Nature.

[12] Prue DM, Martin JE, Hume AS (1980). Behav Ther.

[13] Benowitz NL, Hall SM, Herning RI, Jacob P, Jones RT, Osman AL (1983). N Engl J Med.

[14] Feyerabend C, Bryant AE, Jarvis MJ, Russell MAH (1986). J Pharm Pharmacol.

[15] Jacob P, Benowitz NL (1986). Cotinine Analytical Workshop (EPA).

